# Intra-bone marrow injection of magnesium isoglyrrhizinate inhibits inflammation and delays osteoarthritis progression through the NF-κB pathway

**DOI:** 10.1186/s13018-022-03294-z

**Published:** 2022-08-31

**Authors:** Rong Chen, Xiangwei Li, Zhibo Sun, Junyi Yin, Xiaowei Hu, Jingwen Deng, Xinghui Liu

**Affiliations:** 1 Department of Traumatic Orthopedics, Renmin Hospital, Hubei University of Medicine, Shiyan, 442000 Hubei China; 2 Hubei Key Laboratory of Embryonic Stem Cell Research, Department of Anatomy, School of Basic Medical Sciences, Hubei University of Medicine, No. 30 Renmin South Road, Maojian District, Shiyan, 442000 Hubei China

**Keywords:** Magnesium isoglycyrrhizinate, Osteoarthritis, NF-κB, Intra-bone marrow injection, Cartilage regeneration

## Abstract

**Objective:**

Osteoarthritis (OA) presents cartilage damage in addition to chronic inflammation. However, self-recovery of damaged cartilage in an inflammatory environment is not possible. Mesenchymal stem cells (MSCs) in the bone marrow are a source of regenerative repair of damaged cartilage. To date, whether intra-luminal administration of the bone marrow can delay the progression of OA is still unknown. This study, therefore, aimed to explore the role of intra-bone marrow injection of Magnesium isoglycyrrhizinate (MgIG) in delaying the OA progression and to investigate the underlying mechanism.

**Methods:**

Rabbit OA models were established using the anterior cruciate ligament transection method while a catheter was implanted into the bone marrow cavity. 1 week after surgery, MgIG treatment was started once a week for 4 weeks. The cartilage degradation was analyzed using hematoxylin–eosin staining, Masson’s trichrome staining and Alcian blue staining. Additionally, the pro-inflammatory factors and cartilage regeneration genes involved in the cartilage degeneration and the underlying mechanisms in OA were detected using enzyme-linked immunosorbent assay, quantitative real-time PCR (qRT-PCR) and Western blotting.

**Results:**

The results of histological staining revealed that intra-bone marrow injection of MgIG reduced degeneration and erosion of articular cartilage, substantially reducing the Osteoarthritis Research Society International scores. Furthermore, the productions of inflammatory cytokines in the bone marrow cavity and articular cavity such as interleukin-1β(IL-1β), IL-6, and tumor necrosis factor-α (TNF-α) were inhibited upon the treatment of MgIG. At the same time, the expression of alkaline phosphate, tartrate-resistant acid phosphatase-5b (TRAP-5b) and C-telopeptides of type II collagen (CTX-II) in the blood also decreased and was positively correlated. On the contrary, cartilage-related genes in the bone marrow cavity such as type II collagen (Col II), Aggrecan (AGN), and SRY-box 9 (SOX9) were up-regulated, while matrix metalloproteinase-3 (MMP-3) was down-regulated. Mechanistically, MgIG was found to exert an anti-inflammatory effect and impart protection to the cartilage by inhibiting the NF-κB pathway.

**Conclusion:**

Intra-bone marrow injection of MgIG might inhibit the activation of the NF-κB pathway in the progression of OA to exert an anti-inflammatory effect in the bone marrow cavity and articular cavity, thereby promoting cartilage regeneration of MSCs in the bone marrow, making it a potential new therapeutic intervention for the treatment of OA.

## Background

Osteoarthritis (OA), characterized by joint cartilage destruction, joint inflammation, and degeneration of the subchondral bone, is one of the most common chronic degenerative musculoskeletal disorders with a high disease burden and no available treatments to delay the progression of the disease[1]. Osteoporosis (OP), characterized by excessive bone resorption, microstructural degeneration of bone tissue, and increased risk of fracture due to reduced bone strength, is another common chronic degenerative skeletal musculoskeletal disease[2], while chronic inflammation is the main driver of bone resorption[3]. Although the pathogenesis of OA is unclear, its main pathological features is also a series of inflammatory cascade reactions that lead to degeneration of articular cartilage[4]. Therefore, there may be an inflammatory-related link between OA and OP. In inflammatory joint disease, bone marrow mesenchymal stem cells (BM-MSCs) migrate to the lesion site, aggravating local inflammation and promoting disease progression[5], while senescent BM-MSCs in OA have a decreased ability to differentiate into chondrocytes[6]. Besides, OP leads to bone loss in subchondria, inhibits cartilage differentiation of BM-MSCs, and exacerbates OA progression[7, 8]. Thus, inhibition of inflammation in the bone marrow cavity improves subchondral bone resorption and promotes cartilage differentiation of BM-MSCs, therefore serving as a potential method for treating OA. However, there are few reports on the treatment of OA by intra-luminal administration of the bone marrow.

Increasingly studies have demonstrated the release of pro-inflammatory cytokines such as the interleukin-1β(IL-1β), IL-6, and tumor necrosis factor-α (TNF-α) from the cartilage, synovium and bone, significantly contribute to the OA development[9, 10]. Furthermore, an excessive IL-1β triggers various pro-inflammatory cytokines and catabolic factors, as IL-6, TNF-α, matrix metalloproteinases (MMPs), thereby promoting degradation of the extrachonocyte matrix (ECM), which ultimately leads to OA progression[11]. The Nuclear factor-κB (NF-κB) is an important transcription factor in the inflammatory response, which plays a central role in OA progression by regulating chondrocyte catabolism and synovial inflammation[12], and plays a key role in OP progression by influencing osteoblast function and bone formation[13]. Thus, suppressing the activation of the NF-κB signaling pathway inhibits the inflammatory response and protects the cartilage, therefore serving as a potential method for treating OA.

Magnesium isoglyrrhizinate (MgIG) is the fourth generation of glycyrrhizitic acid preparation derived from traditional Chinese medicine licorice, which is often used clinically in China for the treatment of inflammatory liver disease[14, 15], and is a safe and well-tolerated intravenous drug[16]. Recently, a growing body of literature has demonstrated that MgIG has anti-inflammatory, antioxidant and anti-apoptotic effects [17–20], and its anti-inflammatory mechanism may be through inhibition of the NF-κB pathway[17, 21]. However, the ability of MgIG to exert anti-inflammatory effects in the joint or bone marrow cavity through the NF-κB pathway remains unclear.

Therefore, the present study aimed to explore whether intra-bone marrow injection of MgIG can inhibit intra-luminal inflammation in the bone marrow, further inhibiting intra-articular inflammation, and thus delaying cartilage degeneration. Additionally, it also explored the anti-inflammatory and cartilage regenerative effect of MgIG by inhibiting the NF-κB pathway in the rabbit OA model established by anterior cruciate ligament transection (ACLT). Our research may provide new insights into the anti-inflammatory and cartilage protective effects of MgIG and useful information for developing new strategies to treat OA.

## Materials and Methods

### Reagents

The enzyme-linked immunosorbent assay (ELISA) kits for IL-1β, IL-6, TNF-α and C-telopeptides of type II collagen (CTX-II) and tartrate-resistant acid phosphatase-5b (TRAP-5b) test kit were purchased from SinoBestBio. The TRIzol reagent was procured from Invitrogen while the PrimeScript RT reagent Kit and the qPCR SYBR® Green Master Mix were purchased from TaKaRa Bio. Besides, the BCA protein assay kit and alkaline phosphate (ALP) test kit were procured from Beyotime Biotechnology. The primary antibodies against type II collagen(Col II), MMP-3, p-p65, p65 and glyceraldehyde-3-phosphate dehydrogenase (GAPDH) were procured from HuaAn Biotechnology. Magnesium isoglycyrrhizinate (MgIG; purity 99.3%) was obtained from Chia-tai Tianqing Pharmaceutical Co., Ltd.

### Establishing rabbit OA models and implanting the catheter into the bone marrow cavity

The 6-week-old male New Zealand rabbits (body weight: 2.7–3.2 kg) were procured from the Animal Experimental Center of the Hubei University of Medicine. Experimental techniques are approved by the Animal Care and Use Committee of Hubei Medical University and follow the Guidelines for Animal Research: Guidelines for In vivo experiment reports. Twenty-four rabbits were randomly divided into four groups (six rabbits in each group): the sham group, OA group, OA + MgIG (MC) group (injection of 500 μl 2.5 mg/ml MgIG in the marrow cavity), and OA + MgIG (AC) group (injection of 500 μl 2.5 mg/ml MgIG in the articular cavity). The dose of MgIG was determined by referring to relevant literature[21] and taking into consideration the dose translation from cells to animals. The rabbit OA model was established according to the instructions previously reported by our team members[22]. Briefly, the rabbits were anesthetized with an injection of 3% pentobarbital at auricular vein, and then the right joint capsule was longitudinally opened to expose the anterior cruciate ligaments (ACL),followed by a rupture of the ACL. Meanwhile, the rabbits in the sham group were subjected to only arthrotomy. Under the protection of the sleeve, a 2.0 mm drill was drilled in the OA + MgIG (MC) group on the medial femur (the upper edge of the patella), and then the catheter was implanted into the bone marrow cavity and fixed.1 week after surgery, rabbits in the treatment group were injected with MgIG once a week (Monday), while rabbits in the OA group were injected with the same dose of normal saline. Antibiotics (400,000 units of penicillin per kilogram of body weight) were intramuscularly injected 30 min before surgery and continued for 3 days after surgery. All rabbits were sacrificed 5 weeks postoperatively.

### Measurement of IL-1β, IL-6, TNF-α, ALP, TRAP-5b and CTX-II

The articular cavity effusion and blood were collected according to the instructions we previously reported[23]. Medullary aspirate was collected according to the previously reported instructions[24]. The collected samples were immediately centrifuged at 3000 rpm for 15 min and the supernatants were collected for further analysis. The concentrations of the IL-1β, IL-6, TNF-α and CTX-II in the sample supernatant were measured using commercial ELISA kits following the manufacturer's instructions. The ALP and TRAP-5b levels in the sample supernatant were measured using test kits following the manufacturer's instructions.

### Quantitative real-time PCR

The total RNA was extracted from medullary aspirate and synovium tissues using the TRIzol reagent according to the manufacturer's instructions. cDNA was formed after reverse transcription using the HiScript® III-RT SuperMix for the quantitative real-time PCR (qRT-PCR) kit where the *IL-1β, TNF-a, MMP-3,Col II, Aggrecan(AGN) and SRY-box 9 (SOX9)* genes (Table1) were PCR-amplified using specific primers as well as the SYBR Green/ROX qPCR Master Mix. Expression of GAPDH was used as an endogenous control and the reactions were carried out in a CFX96 qRT-PCR detection system (Bio-Rad, USA) to monitor fluorescence during each PCR cycle of the annealing step. The specific amplification of gene-of-interest was validated by the melting curve. The threshold cycle (CT) values were calculated based on the amplification curves, and the relative expression of the target genes was calculated using the 2^−ΔΔCт^method.

**Table 1:** Primers used for assessing the gene expressions using the qRT-PCR

Gene	Forward (sequence 5′- 3′)	Reverse (sequence 5′- 3′)
Rabbit *IL-1β*	GCC GAT GGT CCC AAT TAC AT	ACA AGA CCT GCC GGA AGC T
Rabbit *TNF-α*	TCT AGT CAA CCC TGT GGC CC	GCC CGA GAA GCT GAT CTA AG
Rabbit *MMP-3*	GCC AAG AGA TGC TGT TGA TG	AGG TCT GTG AAG GCG TTG TA
Rabbit *Col II*	AGTCTTGCCCCACTTACC	TCCCAGAACATCACCTACCA
Rabbit *AGN*	CCACAGACCCTAAGCCTTCT	CCACAGACCCTAAGCCTTCT
Rabbit SOX-9	GGTGAAGGTGGAGTAGAGGCT	GAACGCACATCAAGACGGA
Rabbit *GAPDH*	GGAGGCAGGGATGATGTTCT	TGTTTGTGATGGGCGTGAA

### Western blot analysis

The total proteins from medullary aspirate and synovium tissues were isolated using the radioimmunoprecipitation assay (RIPA) lysis buffer containing protease inhibitors and phosphatase inhibitors. The protein concentrations were calculated using BCA protein concentration determination method. For each sample, the total proteins (30 μg) were loaded and separated by a 10% SDS-PAGE followed by a transfer onto the polyvinylidene difluoride (PVDF) membrane. After blocking with a 5% nonfat milk at 25 °C for 1 h, the membranes were incubated with a primary antibody at 4 °C overnight against Col II (1:1000), MMP-3(1:800), p-p65 (1:1000), p65 (1:1000) and GAPDH (1:5000). After washing three times with TBST, the membranes were incubated with an HRP-conjugated secondary antibody (1:10,000) at 25 °C for 1 h. Finally, the protein bands were visualized with the ECL reagent using the Imaging System (Bio-Rad, USA).

### Histological analysis

The knee samples were fixed in 4% paraformaldehyde at 4 °C for 24 h and then decalcified in 10% EDTA (pH 7.4) for 3 weeks. Subsequently, the joint tissues (the medial femoral condyle) were embedded in paraffin and the 5 μm of the paraffin-embedded tissues were stained using hematoxylin–eosin staining (HE), Masson’s trichrome staining (Masson) and alcian blue staining(Alcian blue). Slides were examined using an optical microscope equipped with a digital CCD camera (Olympus, Japan). The severity of the degradation of the articular cartilage was scored according to the Osteoarthritis Research Society International (OARSI) scoring system as previously described[25].

### Statistical analysis

All the data were analyzed using the statistical software SPSS version 25.0 (IBM Corporation, USA). The data were represented as mean ± standard deviation (SD). The two-way ANOVA and one-way ANOVA were used for analyzing the data, and the homogeneity of variance using the LSD method, the missing variance using the Dunnett-t method. The correlation of each indicator was determined by Pearson linear correlation analysis. The differences were considered significant when the *P*-value < 0.05.

## Results

### Constructing rabbit OA model and implanting a catheter into bone marrow cavity

**Fig. 1:**
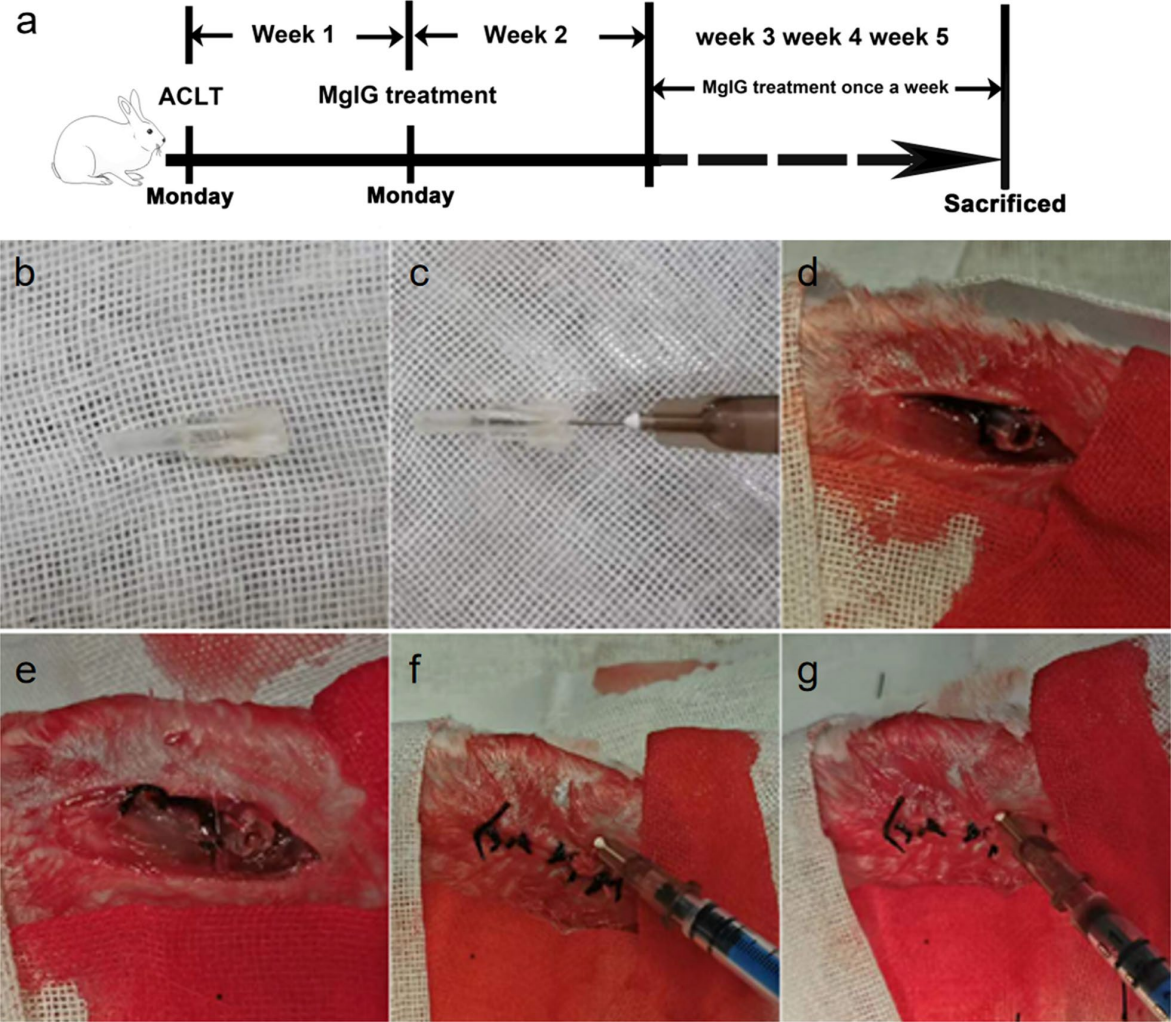
Constructing rabbit OA model and implanting a catheter into bone marrow cavity. **a** The timeline of the experimental protocol. **B, c** two-end catheters of different sizes. **d** Construct an OA model and implant a catheter into the bone marrow cavity at the same incision. **e** Fix the catheter. **F, g** Percutaneous puncture and verification

To facilitate intra-bone marrow injection of MgIG, we used the ACLT method to construct a rabbit OA model and implanted a catheter into the bone marrow cavity. The postoperative recovery and diet in all the groups were normal and the incisions healed well without inflammatory reactions. In addition, the walking of rabbits in the OA + MgIG (MC) group showed no obvious abnormalities. Figure 1a depicts the timeline of the experimental protocol. We used two-end catheters of different sizes (Fig. 1b, c). After constructing an OA model, a catheter was implanted into the bone marrow cavity at the same incision (Fig. 1d). The catheter was then fixed and the skin was sutured to embed the catheter under the skin (Fig. 1e). Finally, the percutaneous puncture was performed and withdrawn to verify that it was in the bone marrow cavity (Fig. 1f, g).

### MgIG attenuates the cartilage damage in a surgical-induced rabbit OA model

**Fig. 2:**
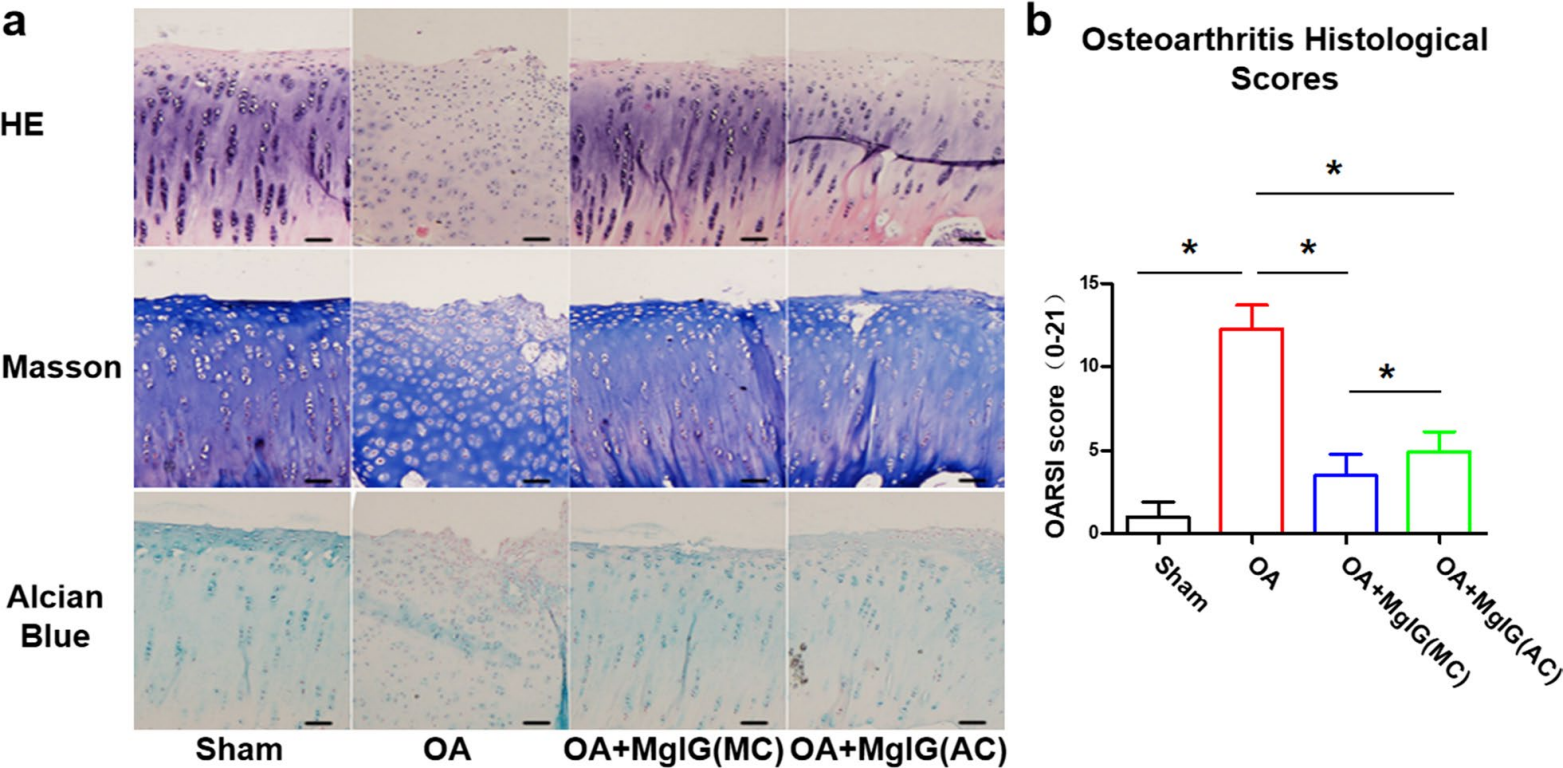
MgIG attenuates the cartilage damage in a surgical-induced rabbit OA model. **a** Cartilage degradation (the medial femoral condyle) was assessed by HE staining, Masson’s trichrome staining and Alcian blue staining. Scale bar: 50 µm. **b** The pathological changes of OA were evaluated using the OARSI score. *P < 0.05. *n* = 6

To investigate the potential role of intra-bone marrow injection of MgIG in OA, MgIG treatment was started 1 week after surgery and intra-articular injection was used as a positive control group. As shown in Fig. 2a, HE staining depicted that the joint surface of the sham group was smooth and the chondrocyte morphology was maintained well, while the presence of chondrocytes in the OA group was irregular and disordered to varying degrees. Moreover, the OA group showed collagen fibrosis disorders in Masson’s trichrome staining, and decreased proteoglycan density in Alcian blue staining, whereas the MgIG treatment group showed good collagen fiber alignment and higher proteoglycan density. Interestingly, MgIG treatment reduces degeneration and erosion of articular cartilage, and the OARSI score confirms that this effect in the OA + MgIG (MC) group was found to be more conspicuous than that in the OA + MgIG (AC) group (Fig. 2b).

### MgIG inhibits inflammation and promotes the ability to differentiate cartilage in the bone marrow cavity

**Fig. 3:**
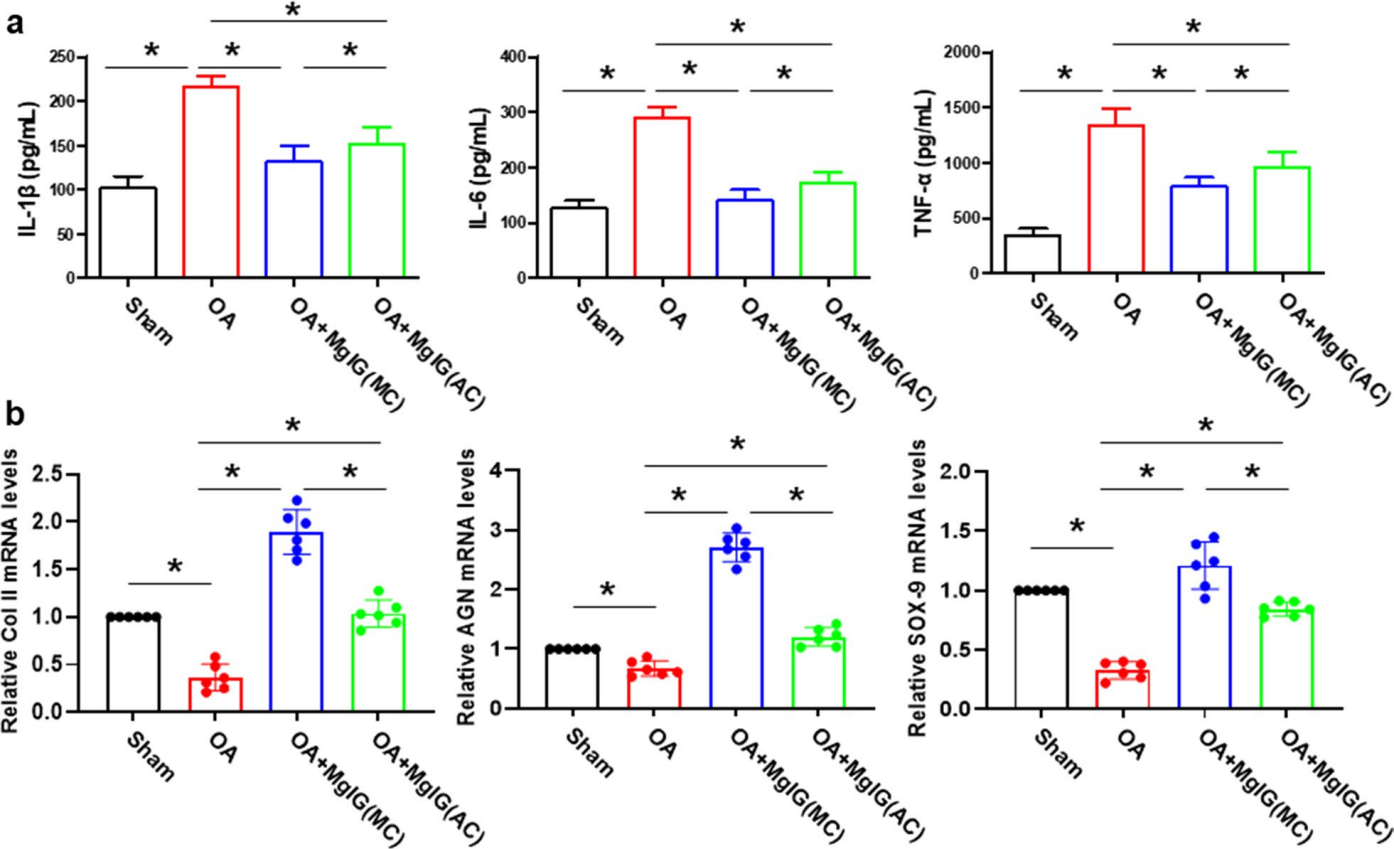
MgIG inhibits inflammation and promotes the ability to differentiate cartilage in the bone marrow cavity. **a** The expression of IL-1β, IL-6 and TNF-α was detected by ELISA. **b** the qRT-PCR was performed for measuring the mRNA expression of *Col II, AGN* and *SOX-9*. *P < 0.05. n = 6

To verify the anti-inflammatory effect of MgIG in the bone marrow cavity**,** ELISA method was performed to detect the inflammatory factor (IL-1β,IL-6 and TNF-α) of the supernatant in the bone marrow cavity. In addition, we used qRT-PCR to analyze chondrogenesis-related genes (*Col II,AGN* and *SOX-9*) to assess the cartilage regeneration capacity of BMSCs under MgIG treatment. As shown in Fig. 3a, the expression levels of IL-1β,IL-6 and TNF-α were down-regulated upon treatment with MgIG, especially in the intra-bone marrow injection group (OA + MgIG (MC)). Meanwhile, the qRT-PCR results indicated that MgIG could significantly promote the expression of *Col II, AGN* and *SOX-9* genes in the OA + MgIG (MC) group (Fig. 3b).

### MgIG inhibits inflammation in the articular cavity

**Fig. 4:**
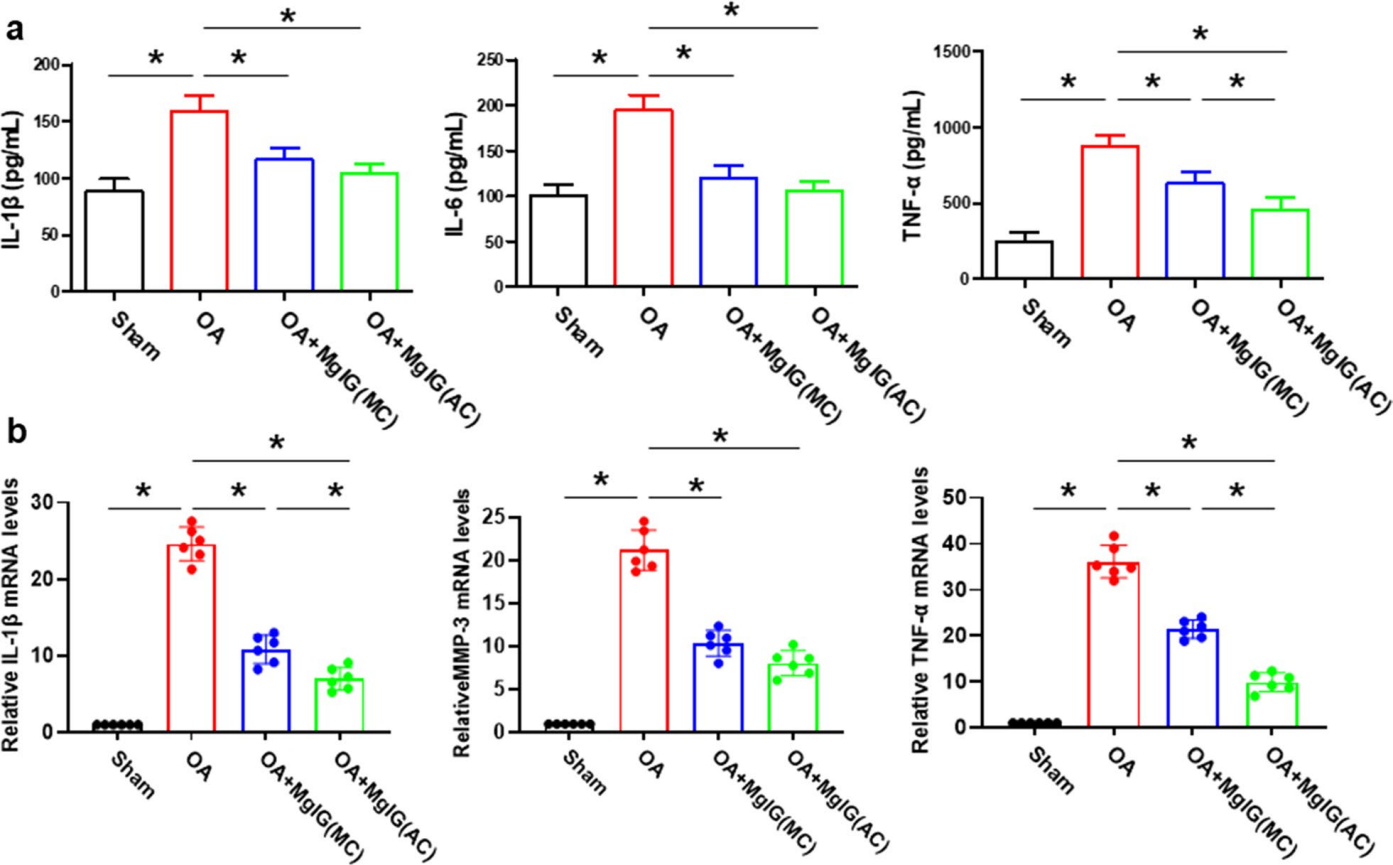
MgIG inhibits inflammation in the articular cavity. **a** The expression of IL-1β, IL-6 and TNF-α was detected by ELISA. **b** the qRT-PCR was performed for measuring the mRNA expression of *IL-1β,MMP-3* and *TNF-α*. *P < 0.05. *n* = 6

To investigate the effects of MgIG on inflammation in the joint cavity, we performed ELISA and qRT PCR analyses to detect the expression of inflammatory factors (IL-1β,IL-6 and TNF-α) in the articular cavity effusion and synovial inflammatory-related genes (*IL-1β,TNF-α* and *MMP-3*), respectively. The results of ELISA and qRT PCR showed that MgIG treatment inhibited intra-articular inflammatory expression, which was evident in the OA + MgIG (AC) group (Fig. 4a, b).However, there were no statistically significant differences in the expression of IL-1β and IL-6 in the articular effusion between the OA + MgIG (AC) and OA + MgIG (MC) groups. Moreover, the expression of MMP-3 was consistent with the above results(Fig. 4b).

### MgIG may exert anti-inflammatory effects by inhibiting the NF-κB pathway

**Fig. 5:**
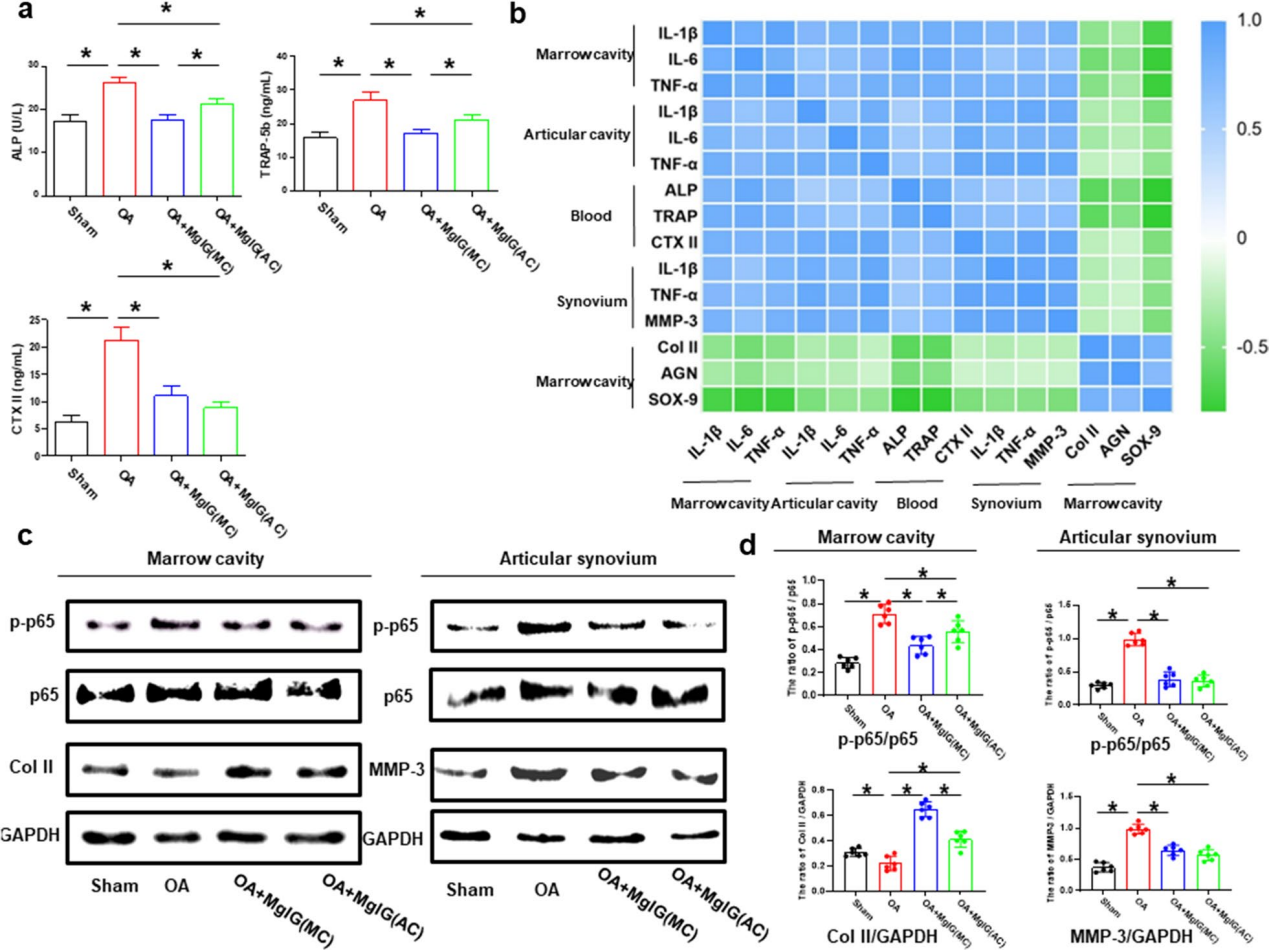
MgIG may exert anti-inflammatory effects by inhibiting the NF-κB pathway. **a** The expression of ALP, TRAP-5b and CTX-II was detected by test kits. **b** Heat map of the correlation of various indicators. **c**, **d** The western blot detected and quantitated the expression of Col II, MMP-3, p65 and p-p65. * *P* < 0.05. *n* = 3

TRAP and ALP expression in the blood reflects bone resorption, while CTX-II reflects cartilage destruction. So, we used test kits to detect ALP, TRAP-5b and CTX-II in the blood. As described in Fig. 5a, MgIG treatment reduces the expression of ALP, TRAP-5b and CTX-II in the blood. Moreover, the expressions of ALP and TRAP-5b were the lowest in the OA + MgIG (MC) group (Fig. 5a), positively correlated with inflammation (IL-1β,IL-6 and TNF-α) and negatively associated with cartilage-related genes (*Col II,AGN* and *SOX-9*) in the bone marrow cavity (Fig. 5b).The expression of CTX-II was lowest in the OA + MgIG (AC) group (Fig. 5a) and was positively correlated with inflammation (IL-1β,IL-6 and TNF-α) in the articular cavity (Fig. 5b).As shown in the heat map in Fig. 5b, the inflammatory expressions in the bone marrow were positively correlated with intra-articular inflammatory expressions and the expression of ALP, TRAP and CTX-II in the blood. Consistent with the above result, the results of western blotting analysis revealed the expression of Col II in the bone marrow cavity was attenuated and the expression of MMP-3 in the articular cavity was increased in the OA group, while treatment with MgIG was found to reverse these trends (Fig. 5c, d). Furthermore, to verify the underlying mechanism by which MgIG exerted an anti-inflammatory effect, NF-κB signaling molecules (p65), a key regulator of inflammation, were detected. As shown in Fig. 5c, d, the p-p65 in the OA group was up-regulated either in the bone marrow cavity or in the articular cavity, which could be suppressed after MgIG treatment.

## Discussion

In addition to chronic inflammation, OA also has cartilage destruction defects, while self-healing of injured cartilage in an inflammatory environment is almost impossible to achieve[10, 26]. However, current studies have confirmed that promoting cartilage differentiation of BM-MSCs has the potential to heal cartilage damage[27, 28]. Although BM-MSCs with a variety of cell differentiation abilities in the bone marrow have the ability to differentiate cartilage, BM-MSCs may differentiate into other cells to aggravate disease progression in OA or OP[5–7].Moreover, IL-1β inhibited chondrogenesis in the mesenchymal stem cells (MSCs) induced by transforming growth factor-β1 (TGF-β1), which may be mechanism by the activation of NF-κB[29]. Therefore, regulation of intra-medullary inflammation by intra-luminal administration of the bone marrow has the potential to delay OA progression.

TRAP is secreted only by mature osteoclasts (OCs), whose levels reflect OC-mediated bone resorption activity[30].Our previous studies have found levels of TRAP-5b, which is an active component of TRAP, increased in joint fluid after joint trauma[23]. In our study, expressions of ALP, TRAP-5b and CTX-II (type II collagen degradation marker) in the OA group are elevated in the blood, while TRAP-5b and CTX-II are positively correlated. Moreover, OC can destroy the subchondral bone and aggravate cartilage degeneration[31]. Consistent with previous literature reports, our results suggest that the expressions of intra-articular and intra-medullary inflammation (IL-1β,IL-6 and TNF-α) are up-regulated in OA. In addition, Inflammatory factors (such as IL-1β,IL-6 and TNF-α) induce OC differentiation, ultimately leading to arthritis cartilage damage[32–34]. Therefore, intra-articular and intra-medullary inflammation is positively correlated with TRAP-5b and CTX-II.

MgIG is a fourth-generation glycyrrhizitic acid preparation[35], clinically used in the treatment of hepatitis diseases, with anti-inflammatory, antioxidant, anti-apoptotic effects[17–21]. Inflammatory factors (such as IL-1β,IL-6 and TNF-α) that, if over-expressed in the bone marrow cavity, will activate OCs, leading to bone loss[3, 7, 32], and if over-expressed in the articular cavity, will lead to cartilage destruction [4, 9, 10]. Moreover, OCs activation leads to bone resorption of subchondral bone, further exacerbating cartilage degeneration[7, 8]. Thus, regulating the inflammatory response in the bone marrow can also play a role in delaying the progression of OA. In arsenic trioxide-induced cardiotoxicity studies, MgIG was effective in reducing the release of IL-1β,IL-6 and TNF-α[17]. According to our results, intra-bone marrow injection of MgIG inhibits intra-medullary inflammation while inhibiting intra-articular inflammation. Since MgIG can reduce the expression of pro-inflammatory mediators (IL-1β,IL-6 and TNF-α) in LPS-induced RAW264.7 cells[21], MgIG may inhibit inflammation by regulating local macrophages in the bone marrow cavity or the articular cavity. Moreover, we also found that MgIG can promote cartilage-related gene expression of BM-MSCs in the bone marrow cavity and reduce the expression of MMP-3 in the synovium. During OA progression, cartilage-associated gene expression of BM-MSCs is down-regulated, while pro-inflammatory factor expression is up-regulated[5, 6]. Besides, MMPs, as ECM structural degradation enzymes, are found to irreversibly destroy the type II collagen, ultimately resulting in the degradation of the ECM[36].Therefore, intra-bone marrow injection of MgIG may delay the progression of OA by inhibiting inflammation and improving the cartilage differentiation ability of BM-MSCs.

Current studies have demonstrated that the NF-κB pathway is widely involved in the occurrence and development of OA by modulating pro-inflammatory factors[37, 38]. Activation of the NF-κB pathway requires a series of phosphorylations, and eventually the NF-κB dimer (including p65/p50) is transferred to the nucleus and binds to inflammation-related genes, thereby facilitating the translation of pro-inflammatory mediators[37]. MgIG may mitigate the translocation of NF-κB by inhibiting the IKK phosphorylation and IκB-α degradation, thereby inhibiting pro-inflammatory cytokines and mediators in LPS-induced RAW264.7 cells[21]. In our study, MgIG was found to significantly inhibit the activation of NF-κB and reduce inflammation in both the bone marrow cavity and the articular cavity. Although there are still no literature reports of MgIG delaying OA progression by inhibiting NF-κB, glycyrrhizic acid can exert anti-inflammatory and cartilage protection effects through the NF-κB pathway[39]. Moreover, our results suggest that MgIG may down-regulate pro-inflammatory factor expression in the bone marrow cavity by inhibiting the NF-κB pathway, leading to up-regulation of cartilage-associated gene expression of BM-MSCs, which is partially consistent with the study by Wehling et al. [29]. Taken together, these results revealed that intra-bone marrow injection of MgIG possibly exerts anti-inflammatory and promotes cartilage repair by inhibiting the activation of the NF-κB pathway, thereby delaying OA progression.

Although intra-bone marrow injection of MgIG does alleviate the progression of OA, our study has some limitations. First, tracing techniques were used to further verify that intra-bone marrow injection of MgIG can lead to the transfer of cells or proteins to the articular cavity. Secondly, other OP-related indicators need to be detected, such as bone density. Finally, we will design a sustained-release drug carrier for bone marrow drug delivery.

## Conclusions

In the ACLT-induced rabbit OA model, the expressions of pro-inflammatory inflammatory factors in the bone marrow cavity and the articular cavity were elevated and positively correlated with the expression of TRAP-5b and CTX-II in the blood. Our results demonstrated intra-bone marrow injection of MgIG to possibly inhibit the activation of the NF-κB pathway to resist the intra-medullary and intra-articular inflammation in the OA progression, thereby promoting the cartilage differentiation capacity of BM-MSCs, which in turn facilitates the recovery of cartilage injury. Therefore, intra-bone marrow injection of MgIG may be a novel therapy for treating OA.

## Data Availability

The data sets generated and analyzed during the current study are available from the corresponding author on reasonable request.
